# Delivery of Virtual Care in Oncology: Province-Wide Interprofessional Consensus Statements Using a Modified Delphi Process

**DOI:** 10.3390/curroncol28060445

**Published:** 2021-12-13

**Authors:** Matthew C. Cheung, Bryan B. Franco, Nicholas Meti, Alia Thawer, Houman Tahmasebi, Adithya Shankar, Andrew Loblaw, Frances C. Wright, Colleen Fox, Naomi Peek, Vivian Sim, Simron Singh

**Affiliations:** 1 Division of Medical Oncology and Hematology, Department of Medicine, Sunnybrook Health Sciences Centre, Toronto, ON M4N 3M5, Canada; matthew.cheung@sunnybrook.ca (M.C.C.); adithyashankar25@gmail.com (A.S.); vsimbulsim@gmail.com (V.S.); 2 Department of Medicine, University of Alberta, Edmonton, AB T6G 2G3, Canada; bfranco@qmed.ca; 3 Department of Medicine, University of Toronto, Toronto, ON M5S 3H2, Canada; nick.meti@mail.utoronto.ca (N.M.); houman.tahmasebi@mail.utoronto.ca (H.T.); 4 Department of Pharmacy, Sunnybrook Health Sciences Centre, Toronto, ON M4N 3M5, Canada; alia.thawer@sunnybrook.ca; 5 Department of Radiation Oncology, Sunnybrook Health Sciences Centre, Toronto, ON M4N 3M5, Canada; Andrew.loblaw@sunnybrook.ca; 6 Department of Surgery, Sunnybrook Health Sciences Centre, Toronto, ON M4N 3M5, Canada; Frances.Wright@sunnybrook.ca; 7 Ontario Health (Cancer Care Ontario), Toronto, ON M4N 3M5, Canada; colleen.fox@ontariohealth.ca (C.F.); naomi.peek@ontariohealth.ca (N.P.)

**Keywords:** consensus, virtual care, teleoncology, telemedicine

## Abstract

Virtual cancer care (i.e., teleoncology) was rapidly adopted during the COVID-19 pandemic to meet the needs of patients with cancer. However, there is a paucity of guidance for clinicians regarding virtual cancer care. We sought to develop consensus-based statements to guide the optimal provision of virtual care for clinicians caring for patients with cancer, using a modified Delphi consensus process with a 29-member panel consisting of an interprofessional group of clinicians caring for patients with cancer and patient representatives. The consensus process consisted of two rounds and one synchronous final consensus meeting. At the end of the modified Delphi process, 62 of 62 statements achieved consensus. Fifty-seven statements reached consensus in the first round of the process. Concerns regarding the ability to convey difficult news virtually and maintaining similar standards as in-person care without disproportionate strain on clinicians and patients were addressed in the consensus process. We achieved interprofessional consensus on virtual cancer care practices. Further research examining the impact of virtual cancer care on person-centred and clinical outcomes are needed to inform practices during the COVID-19 pandemic and beyond.

## 1. Introduction

The novel coronavirus, SARS-CoV-2, responsible for the coronavirus disease (COVID-19), is the foremost public health priority globally and has dramatically altered the delivery of clinical care. Physical distancing, which was the quintessential public health intervention to limit the spread of the virus, greatly limited traditional in-person cancer care [1]. Persons diagnosed with cancer, as well as those undergoing active cancer treatment, are at higher risk for COVID-19 complications [2,3]. A recent systematic review and meta-analysis found that the case-fatality rate of COVID-19 in patients with cancer was 22% compared with 6% for those without cancer [4]. Despite COVID-19 vaccines becoming more widely available, emerging variants of concern combined with uncertainty about vaccine response require that patients with cancer to balance the competing risks of contracting COVID-19, and the morbidity and mortality associated with cancer [5]. Patients with cancer continue to face unique challenges during the pandemic related to their care, such as physical distancing efforts hindering in-person care; increased risk of infection when attending appointments and treatments; treatment delays or unavailability due to ramp downs of clinical care; management of adverse effects of treatments [6] and suboptimal responses to vaccination [7]. To meet pressing patient needs, virtual cancer care (i.e., teleoncology) has been rapidly adopted to prioritize the safety of this patient population [1,8,9,10,11,12,13,14,15].

Virtual cancer care was originally developed to improve access to care in remote and rural settings, as well as to meet the growing demands by a limited oncology workforce [16]. Virtual care can include consultations, follow-up appointments, remote supervision of chemotherapy, and access to clinical trials [16]. There have been published examples of virtual cancer care for more than 20 years [17], including practices from North America and Australia [16,18,19]. Studies of teleoncology used to meet the needs of rural patients have demonstrated high patient and provider satisfaction [18,20]. A study in Australia found that virtual care allowed for safe remote administration of chemotherapy [21]. In addition, teleoncology initiatives are associated with decreased costs and improved access to care [16]. However, despite these promising studies, there is a dearth of high-quality evidence that demonstrates the impact of virtual cancer care on important person-centred and clinical outcomes or determines the appropriateness and ideal context in which to administer virtual care [22,23].

Although the COVID-19 pandemic has thrusted virtual cancer care to the fore, there are currently no widely established best practices nor evidence-based guidance for clinicians [23]. As an unprecedented number of patients with cancer receive their care remotely, identification of best practices for virtual cancer care is urgently needed to guide clinicians during the COVID-19 pandemic and beyond [24]. With the absence of high-quality evidence during a pandemic that precluded methodologically rigorous and pragmatic studies, the development of guidance for virtual cancer care practice using other feasible methodologies are warranted [23]. Consequently, Ontario Health (Cancer Care Ontario) identified an urgent need to develop consensus practice statements to provide guidance to clinicians. We developed expert consensus recommendations for virtual cancer care using a modified Delphi process with the intention to set the foundation for the future of cancer care in Ontario, a Canadian province with 14.5 million people.

## 2. Materials and Methods

### 2.1. Modified Delphi

We used a modified Delphi consensus process based on guideline development methodology established by the American Society Clinical Oncology [25]. A formal consensus process provides a transparent and reproducible method to develop recommendations when high-quality evidence is lacking, making it suitable in addressing questions about virtual cancer care. To limit in-person meetings during the pandemic, the process was conducted virtually using both an online survey platform (Survey Monkey Inc., San Mateo, CA, USA, www.surveymonkey.com, 5 April 2021) and teleconference software (Zoom Video Communications Inc., San Jose, CA, USA, www.zoom.us, 5 April 2021). This study was approved by the Sunnybrook Health Sciences Centre Research Ethics Board.

### 2.2. Literature Review

To create the first draft of consensus statements, we conducted a systematic literature review of Embase and MEDLINE for peer-reviewed articles in April 2020 (Supplementary Materials, Figure S1). The inclusion criteria were purposely broad, and we included any articles that described or investigated synchronous virtual cancer care delivered by oncology physicians. To ensure that we considered new practice-changing publications that may affect our consensus statements, we incorporated results from a systematic review of randomized control trials in virtual cancer care conducted by Ontario Health (Cancer Care Ontario) in March 2021 [23].

### 2.3. Steering Committee

The Steering Committee (SC) consisted of 10 members: a medical oncologist, hematologist oncologist, radiation oncologist, surgical oncologist, patient representative, oncology pharmacist, one research coordinator, and three medical trainees (a resident physician and two medical students). The SC identified key questions (Appendix A) regarding virtual cancer care and used literature as a guide to draft statements. Clinical questions were grouped into three sections: (A) demographics, logistics, and implementation, (B) diagnosis and prognosis, and (C) clinical characteristics, active management, and follow-up. The SC drafted the first set of consensus statements by using literature to answer key clinical questions (Supplementary Materials, Table S1).

### 2.4. Consensus Process

We assembled a diverse Consensus Group (CG) consisting of an interprofessional collection of clinicians and patients to capture a wide spectrum of perspectives and avoid potential biases inherent in the development of consensus recommendations [25]. Ontario is a province with a population of 14.5 million distributed over a large geographic area and thus we aimed to include individuals with opinions and experiences related to rurality, discipline, Indigenous status, and clinical specialty [26]. Therefore, we invited clinicians, patient representatives, and administrators (including virtual care stakeholders such as the Ontario Telehealth Network) across the province associated with Ontario Health (Cancer Care Ontario) to be part of the CG.

We conducted two rounds of anonymous modified Delphi surveys and a final synchronous virtual consensus meeting. Draft statements were inputted into an anonymous online survey and distributed via email to the CG (Round 1 of the consensus process). We used a ≥75% threshold, determined a priori, for consensus with agreement achieved by a “agree” or “strongly agree” on a 5-point Likert scale. Respondents were able to provide open-text comments for each draft recommendation. After Round 1, the SC used comments to revise statements that did not achieve 75% agreement. The revised statements were sent to the CG for a total of two consensus rounds. Two e-mail reminders were sent to CG members for each round. After Rounds 1 and 2, statements not reaching consensus and those that achieved consensus but had comments with clear suggestions for improvement were presented to the CG at the final consensus meeting, a synchronous virtual discussion. A simple majority via anonymous polling (using Zoom) was used to determine consensus at the final meeting.

## 3. Results

### 3.1. Consensus Group

The CG had twenty-nine members and included medical cancer specialists (four medical oncologists, two hematologists, three radiation oncologists), seven surgical oncologists (including a neurosurgeon), three family physicians, one psychiatrist, two patient representatives, two nursing and allied health professionals, and four health care administrators. CG members included academic and community-based clinicians, as well as representatives from Indigenous, and rural/remote communities. The response rate in Round 1 was 97% (*n* = 28) and 93% (*n* = 27) in Round 2. There were 22 members of the CG present during the final consensus meeting.

### 3.2. Consensus Process

**Figure 1:**
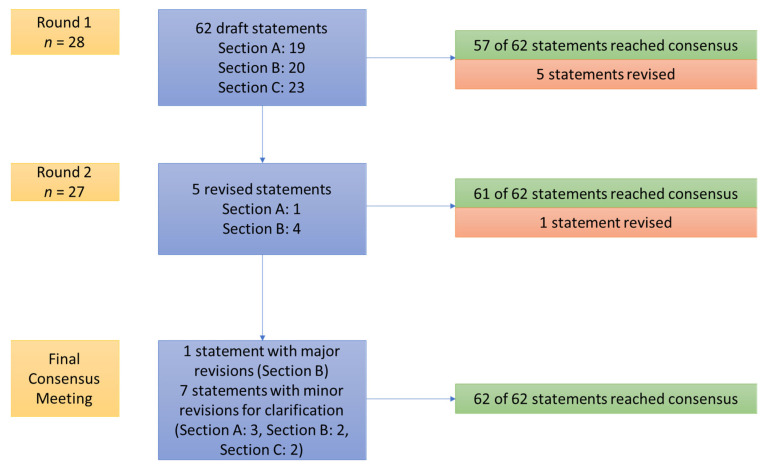
Overview of the consensus process.

The SC drafted 62 statements (Figure 1) from 90 articles identified in the systematic review (no new articles were identified from the more recent systematic review of randomized control trials). After Round 1, 57 of 62 (92%) statements achieved consensus. Statements not reaching consensus were revised according to comments and sent to the CG. After Round 2, 61 of 62 (98%) statements reached consensus. During the final consensus meeting, the statement that did not reach consensus was discussed, revised, and re-rated. Qualitative feedback was summarized and provided to CG members during the final meeting. Seven statements that were revised according to comments related to clarification and wording were also voted upon during the final meeting. There was agreement on all the revised statements after the final consensus meeting.

### 3.3. Section A—Demographics, Logistics, and Implementation

The CG reached consensus regarding recommendations stating that all patients with cancer should be offered virtual care, with particular attention to overcoming accessibility issues (e.g., those with limited comfort with technology, language barriers) (Table 1). Statements surrounding logistics reinforced that documentation should be consistent with in-person care in addition to informed consent for the virtual encounter. Statements about the integration of systems of cancer care, including electronic medical records, interdisciplinary collaboration, access to local health care resources, were addressed in several statements. Finally, the CG reached consensus regarding the need for access to virtual care training options for clinicians and patients.

After Round 1, consensus was achieved on 18 of 19 statements in Section A. in the statement that did not meet consensus (Statement A2f), it (Table 1) had 39% agreement. This statement originally recommended that clinicians use an onboarding process (gradually increasing familiarity with virtual cancer care through using strategies such as selecting engaged, technologically savvy, and familiar patients) when starting to use teleoncology [27]. The comments suggested that the CG perceived that a self-guided onboarding process was not necessary for clinicians. Rather, comments suggested that formal training options in virtual care for clinicians be developed and made available to facilitate the process. This statement was revised accordingly and all statements in Section A reached consensus in Round 2. Minor revisions for clarity were unanimously agreed upon during the final consensus meeting for three Section A statements (A2f, A2g, and A4b). All clarifying revisions were related to making statements more adaptable to different clinical scenarios.

### 3.4. Section B—Diagnosis and Prognosis

The CG reached consensus on 20 statements to guide the communication of diagnostic and prognostic information (Table 2). The recommendations emphasized the importance of understanding patient preferences regarding method of communication before this information is conveyed. In addition, the CG achieved consensus specific to recommendations to optimize communication through virtual platforms. Finally, CG members highlighted the unique situation of conveying bad news such as new metastatic or palliative diagnoses. The need for clinicians to exercise discretion in the decision to convey this information virtually is emphasized in the consensus statements. Specifically, patient-centred factors including the level of support available to the patient, the symptom burden experienced by the patient, and the nature of the patient–provider relationship should be considered in decisions to use virtual cancer care during these encounters.

After Round 1, consensus was achieved on 16 of 20 statements in Section B except B1a (Table 2), which had 61% agreement. This section originally contained recommendations for the administration of a formal health literacy screen, such as the Brief Health Literacy Screen [28], in preparation for the virtual encounter. CG members indicated that difficulties in operationalizing a formal screening tool would outweigh its benefits and this recommendation was removed in the final statement.

Similarly, Statement B2a initially recommended the use of a structured and validated framework to communicate diagnosis and prognosis virtually and reached 57% agreement with many comments suggesting that a formal framework is not necessary. In Round 2, the revised statement, which included wording on the use of empathic communication and consideration of the patient–provider relationship, achieved 62% agreement. During the final consensus meeting, this statement was revised after the CG discussed that the decision to disclose a diagnosis or prognosis virtually should be influenced by whether virtual care can better meet a patient’s needs relative to in-person care (e.g., if virtual care provides more timely access).

Statement B3a, which originally contained wording about challenges in effectively communicating metastatic or palliative diagnoses, had 61% agreement after Round 1 (Table 2). CG members suggested that this is highly dependent on clinical and patient factors and may be feasible for some patients; the statement was revised to reflect consideration based upon the patient–provider relationship, expected patient response to the diagnosis, and available support, and reached consensus in Round 2 with 96% agreement.

### 3.5. Section C—Clinical Characteristics, Active Management, and Follow-Up

All 23 Section C statements reached consensus in Round 1 (Table 3). Two statements (C1c and C4d) were discussed in the final consensus meeting to improve wording based on comments received. There was agreement with statements that acknowledged patient and health care provider preferences, in addition to clinical appropriateness, as important considerations when deciding to provide virtual cancer care. Furthermore, consensus statements addressed situations that may warrant in-person assessment, such as circumstances when physical examination is necessary. Recommendations unique to surgical oncology, radiation, systemic therapy, and survivorship reached agreement. Finally, in addition to active management, there was consensus regarding the use of virtual cancer care other aspects of care (e.g., access to clinical trials, symptom and pain management, cancer prevention).

### 3.6. Qualitative Feedback

We achieved consensus for 62 statements regarding the practice and delivery of virtual oncology care. A few overarching themes emerged from the comments and discussions during the modified Delphi process. First, CG members generally indicated that virtual cancer care should follow the same standards as in-person care, and thus recommendations should reflect current standards of care. Second, CG members felt that health care systems and infrastructure should strive to minimize undue burden on patients and families, and clinicians relative to in-person care. Finally, respondents generally perceived virtual cancer care as appropriate for most patients in most clinical scenarios when physical examination was not deemed necessary. However, recommendations require flexibility so that they can be adapted to different clinical and patient situations.

## 4. Discussion

A diverse CG, consisting of an interprofessional group of clinicians and patient representatives, reached consensus on 62 statements regarding the delivery of virtual cancer care. Almost all statements reached consensus after the first round. Comments provided by respondents and discussion during the final meeting highlighted that the consensus achieved was premised on the perception that virtual cancer care should reflect the same standards of practice as in-person care. Furthermore, most minor clarifications and wording revisions reflected the desire to adapt virtual cancer care according to clinical and patient needs. To our knowledge, this consensus document is the first to focus on the delivery of virtual cancer care.

Although our findings suggest that virtual care is suitable for most patients and clinical scenarios when physical examination was not required, there were areas of initial disagreement related to discussing bad news or difficult clinical scenarios with patients (e.g., a new diagnosis of cancer, recurrent and/or progressive disease, and poor prognosis). Early discussions revolved around whether these scenarios should always prompt an in-person visit, rather than a virtual one. This is consistent with a recent study from Ontario that found physicians’ perceptions on the quality and safety of virtual cancer care improved since its implementation [13]. In addition, published reports on experiences with virtual cancer care prior to the COVID-19 pandemic have suggested that it is possible to successfully deliver bad news virtually after consideration of a patient’s unique context (e.g., available local supports) [29]. In our study, these findings were reflected with the CG achieving consensus after the statements were revised to explicitly consider patients’ unique context (such as the ability to understand, process, and follow-up on health information and the patient–provider relationship) as well as clinical appropriateness. Our understanding of clinical decision making surrounding the suitability of certain patients and difficult clinical scenarios will likely evolve as virtual cancer care continues to be further normalized as part of patients’ cancer journeys.

Another important aspect of virtual cancer care elucidated during the consensus process is the relative lack of infrastructure for both patients and health care providers to provide optimal virtual care to cancer patients (e.g., digital platforms, communications, logistics, and specific administration supports). Moreover, infrastructure varies greatly across different institutions and regions of the province, although professional organizations, such as European Society for Medical Oncology (ESMO) [30], tout virtual cancer care, there is a paucity of guidance regarding systemic changes to integrate it into routine oncology practice [31]. For example, whereas clinician reimbursement for virtual care is necessary, it alone is likely insufficient in supporting virtual cancer care during the COVID-19 pandemic and beyond [1,13]. To this end, our consensus statements on virtual oncology practice, while also providing guidance to clinicians provincewide, can serve as a catalyst and initial guide for institutions, policymakers, and governments when developing system-wide supports for virtual cancer care.

### Strengths and Limitations

Strengths of our consensus process include the diversity of the CG (clinicians, patients, rurality) and the high response rate. Furthermore, the anonymity between rounds in the modified Delphi process prevented overwhelming influence of any particular member [32]. The synchronous final consensus meeting also facilitated clarification on the sources of disagreement and were resolved accordingly. However, our recommendations should be considered in the context of our study’s limitations. Although we used a comprehensive literature review to draft our statements and validated methods to achieve agreement, the statements should be interpreted as expert opinion based on consensus in the broader context of an evolving and rapidly growing field of virtual cancer care.

## 5. Conclusions

Our consensus statements provide clear guidance to clinicians delivering virtual cancer care. Virtual care should meet the same standards expected with in-person cancer care. Patient preferences and circumstances are important factors to consider to appropriately offer and tailor virtual cancer care. With the expansion of virtual cancer care during and after the COVID-19 pandemic, further research is needed to guide best practices. Prior to the pandemic, the impetus for virtual cancer care was to improve access to care (e.g., provide care for patients in rural areas) [23]. However, the paradigm surrounding virtual cancer care has shifted from a tool to improve access to care, to an essential modality in almost all aspects of cancer care. Therefore, research is needed to examine virtual cancer care’s impact on important person-centred and clinical outcomes to inform best practices in the future.

## Figures and Tables

**Table 1:** Section A—Demographics, implementation, and logistics.

Consensus Statement	Agreement		
	Round 1 (*n* = 28)	Round 2 (*n* = 27) *	Consensus Meeting *
Demographics and Implementation			
A1a. All patients should be considered and, if clinically feasible, offered the option of virtual cancer care regardless of demographics (e.g., gender, race/ethnicity, language spoken, income, education, rurality, physical and/or mental disabilities, indigenous identity). Special effort should be made towards patients without good access to technology, or those who are uncomfortable with using technology.	89%	-	-
A1b. It is recommended that resources be created and disseminated to all health care providers and patients to overcome barriers to virtual cancer care. These can include written, video, and/or verbal guidance provided by a member of the oncology team (e.g., clinical administrator) in advance of the virtual visit or point of care resources.	96%	-	-
A1c. Efforts should be made to ensure that virtual cancer care systems are made as easily accessible as possible. For example, health care providers and/or patients who may not have easy access to computer/internet platforms should be provided the option for a telephone visit instead, where appropriate.	96%	-	-
A1d. One suboptimal or unsuccessful technology encounter does not exclude a patient from future technology encounters as long both patient and provider deem clinically and logistically feasible.	89%	-	-
A1e. Caregivers are encouraged to attend virtual visits, especially for patients with language barriers, self-reported lack of comfort with teleoncology, hearing impairment, or cognitive impairment, it may be helpful to organize a family member to be on the teleoncology encounter at the same time. Health care providers should ensure patient privacy and consent is obtained to discuss details of their care with additional persons.	96%	-	-
A1f. A pre-determined and dedicated time period should be allocated for virtual visits. Both health care providers and patients should ensure an environment that is distraction free and provides confidentiality.	96%	-	-
A1g. Adequate time for health care providers prior to and following a virtual care visit should be planned as additional steps (e.g., electronic requests for outside labs, imaging, prescriptions) may increase the amount of time required per visit.	93%	-	-
Equipment and Environment			
A2a. Health care providers should have access to reliable internet connection and an electronic device (e.g., computer, tablet, or smartphone) if using video technology for virtual care.	100%	-	-
A2b. Back-up systems, such as telephone (landline or cellular), should be available during virtual care visits, should technical difficulties arise. Landline telephone is preferred for call quality/stability, if available. If not, then cellular/mobile phone can be used.	96%	-	-
A2c. All visits should be documented using the same standards as in-person assessment.	96%	-	-
A2d. Documentation should state that the visit was carried out virtually and that the patient has consented to a virtual assessment, understanding the limitations of virtual visits, including lack of physical examination.	96%	-	-
A2e. Electronic medical record systems that allow health care providers to access system-wide investigations (including biochemical, radiological, pathological data—that may have been completed outside the institution) and relevant documentation are critical to facilitate virtual visits with patients.	93%	-	-
A2f. To optimize the delivery of virtual cancer care, health care providers and patients should have access to training options (e.g., teleoncology modules and programs). This training should be supported and disseminated by institutions, provincial entities, and/or in collaboration with other virtual cancer care stakeholders (e.g., Ontario Telehealth Network).	39%	78%	100%
A2g. If video-based technologies are not available and/or if telephone communication is preferred by health care providers and/or patients, then telephone communication may be reasonable.	89%	-	83% (*n* = 18)
Collaborative and Interdisciplinary Care			
A3a. Multidisciplinary tumour boards and case conferences involving medical oncologists, surgeons, radiation oncologists, general practitioners, radiologists, pathologists, nursing, pharmacists, and allied health professionals are feasible and should remain standard of care for discussing cancer patients. Confidential and secure platforms should be chosen to host conferences and discussions as per standards.	93%	-	-
A3b. Involvement of local health care providers in teleoncology encounters should be supported, if possible and available. Administrative support may be required.	93%	-	-
Local Health Care Resources			
A4a. If a care plan is initiated via virtual platforms, a health care provider must be available at the treatment centre to guide and support treatments (e.g., chemotherapy and infusion reactions).	82%		
A4b. Delivery of virtual cancer care should include efforts to link patients with local laboratory (i.e., blood test) and/or imaging services when appropriate. However, test results should be available to the health care provider and comparable to previous investigations (e.g., comparing imaging scans at follow up visits). If not available, then testing should be carried out at the health care provider’s institution. In order to ensure this is completed in an efficient manner, administrator support is encouraged.	75%	-	100% (*n* = 18)
A4c. Access to primary care and emergent care must be included in the discussion of risks and benefits of virtually managed cancer care. Patients receiving virtual cancer care should be counselled on possible risks specific to their care (e.g., chemotherapy toxicity, lymphedema, post-surgical complications) and cancer (e.g., visceral crisis) and appropriate avenues to reach care. Therefore, we encourage that the patient’s local health care provider is made aware of ongoing cancer care and that patients are aware of local resources in the event of complications.	93%	-	-

* Percentages are included if the statement was presented to the Consensus Group.

**Table 2:** Section B—Diagnosis and prognosis.

Consensus Statements	Agreement		
	Round 1 (*n* = 28)	Round 2 (*n* = 27) *	Consensus Meeting *
Preparation			
B1a. Prior to and during virtual cancer care visits (especially initial consultation), health care teams are encouraged to assess the patient’s ability to understand, process and follow up on the communication of health information delivered virtually (digital and/or over the telephone).	61%	81%	100% (*n* = 18)
B1b. When clinically appropriate, patient preferences regarding method of communication (phone, videoconference, in person) to hear diagnostic and prognostic information should be understood by the health care provider before diagnosis/prognosis is conveyed. Moreover, effort should be made to have a caregiver present, depending on patient preference.	100%	-	-
B1c. When there is uncertainty about definitive diagnosis and/or prognosis, collaborating amongst health care providers/disciplines should occur prior to telecommunication with the patient such that a clear plan of care can be shared virtually.	82%	-	-
B1d. If after collaboration uncertainty is still present, a clear plan should be constructed to communicate to the patient how this uncertainty will be clarified. This plan can be communicated virtually to the patient by one or more health care providers involved.	82%	-	-
Communication			
B2a. The discussion of initial cancer diagnosis and prognosis may occur over virtual care platforms, if that would meet the needs of the patient (e.g., more timely discussions, better family support or patient inability to travel). *In addition to in-person standards of communication (e.g., ensuring caregiver and/or supports are available), key elements of an effective virtual interaction regarding cancer diagnosis include (but are not limited to):*	57%	67%	91% (*n* = 22)
B2b. Use of video over telephone, if available	79%	-	-
B2c. Placement of camera should be at eye level so that the health care provider does not appear above the patient.	75%	-	-
B2d. Explain at the outset that the conversation is about diagnosis and next steps.	86%	-	-
B2e. Introduce all health care providers present and ask for an introduction of all family/friends that are part of the virtual conversation.	100%	-	-
B2f. If using video-based platforms (that allow sharing digital information on the screen) to conduct a virtual visit, virtual aids that complement the discussion (e.g., imaging, pathology reports, prediction tool outputs) can be shared with the patient, depending on patient preference and feasibility.	71%	100%	-
B2g. Allow for pauses for question asking and answering.	96%	-	-
B2h. Confirm understanding using the teach back method (i.e., ask patient to explain plan back to the health care provider).	93%	-	-
Communication of Treatment Plans			
B2i. Plan for the interaction and have information on hand that you anticipate patients may ask (e.g., avenues for treatment access, potential start dates, treatment delivery site).	100%	-	-
B2j. Discuss and execute referrals to other services dependent on patient need and treatment plan, e.g., social work, nursing, pharmacy, drug reimbursement or dietician.	100%	-	-
B2k. Include prognosis information as part of the discussion in accordance with patient preference.	100%	-	-
B2l. All interactions about diagnosis and prognosis should be supplemented with educational material (e.g., drug information sheets, disease information, written care plan), and an avenue (e.g., incoming phone line, patient portal, follow up appointment, email) should be provided for questions after review of information and literature.	86%	-	94% (*n* = 18)
New diagnoses and prognoses with anticipated limited life expectancies			
B3a. The health care provider should exercise discretion as to whether to convey a metastatic/palliative diagnosis virtually or in-person. The provider should consider the nature of the patient/provider relationship, the expected response to the metastatic/palliative diagnosis, as well as the level of support available to the patient. *Exceptions to this statement may include:*	61%	96%	-
B3b. Situations where there is urgency to initiate treatment and virtual care facilitates expediency.	86%	-	-
B3c. The patient has a high symptom burden where they cannot physically attend appointment.	96%	-	-
B3d. A virtual interaction enables a local health care provider to be part of the interaction, where they will be key in co-managing patients and treatment moving forward.	79%	-	-

* Percentages are included if the statement was presented to the Consensus Group.

**Table 3:** Section C—Clinical characteristics, active management, and follow-up.

Consensus Statements	Agreement		
	Round 1 (*n* = 28)	Round 2 (*n* = 27) *	Consensus Meeting *
General Clinical Considerations			
C1a. If physical examinations and/or investigations (e.g., bloodwork, imaging, pathology) essential for diagnosis/prognosis, symptom management, and/or choice of treatment could not be obtained through a virtual consult, an in-person face-to-face consult is required.	100%	-	-
C1b. Appropriateness of engaging individual patients in virtual care visits for active management depends on the health care provider, as well as patient preference when clinically appropriate.	89%	-	-
C1c. Both curative and non-curative intent virtual management of patients with cancer is appropriate, unless in-person assessment is required by either the health care provider or the patient.	93%	-	88% (*n* = 17)
C1d. When available (depending on treatment centre infrastructure), allied health care (e.g., nursing, pharmacy, social work) support should be offered to ensure optimal patient care.	96%	-	-
C1e. General practitioners who actively follow your patients with cancer should be engaged in the virtual discussions, if possible and appropriate, in order to facilitate optimal longitudinal patient care.	75%	-	-
C1f. Health care providers using virtual assessment tools should ensure patients are assessed at the same frequency of visits as in-person assessments.	82%	-	-
C1g. Patients assessed virtually should still be referred for clinical trial eligibility where appropriate.	100%	-	-
C1h. Virtual care could be used for cancer prevention, symptom and pain management, and the assessment of nutrition, drug toxicity, and psychosocial factors (e.g., psychological counselling, activities of daily living, etc.).	100%	-	-
C1i. Virtual follow-up visits that require discussion about recurrent or progressive disease and/or change of treatment due to treatment failure should prompt the health care provider to request that the patient be accompanied on the telephone or video conference by a caregiver.	75%	-	-
Surgical Oncology			
C2a. First consultations with potential surgical cancer patients should be held in-person if there is a requirement for formal physical examination of relevant organ system or other in-person investigations. Otherwise, virtual consult is appropriate.	96%		
C2b. Surgical planning and post-operative follow up of patients with cancer could be conducted virtually, either when no additional physical examinations or investigations (e.g., bloodwork, imaging, pathology) are needed, or when these examinations and investigations could be completed locally for patients living in remote areas. In the latter case, such consultations are only appropriate if the surgeon is comfortable with the extent of physical examinations performed by local health care providers and their experiences and skills.	100%	-	-
C2c. Post-surgical patients can be assessed virtually unless the need for wound assessment and/or physical examination is required to provide optimal care. If virtual care is performed, we recommend engaging in homecare to patients with active wound issues (i.e., wound care).	82%	-	-
Radiation Therapy			
C3a. First consultations with potential radiation oncology cancer patients should be held in person if formal physical examination of relevant organ system is necessary. Otherwise, virtual care is appropriate.	86%	-	-
C3b. Patients on surveillance or observation following definitive radiation therapy with curative intent can be followed virtually, unless symptoms arise on review of systems that trigger an in-person assessment. Engagement of allied health and family health care providers is recommended.	93%	-	-
C3c. Discussion of radiation treatments can be conducted virtually, as long as there is no requirement for an in-person assessment.	96%	-	-
Medical Therapy			
C4a. First consultations with potential medical and hematology oncology cancer patients should be held in-person for formal physical examination of relevant organ systems and/or any pre-treatment procedures, if necessary. If not, then virtual assessment is appropriate.	86%	-	-
C4b. Patients with cancer receiving active systemic anti-cancer therapy (intravenous and/or oral) can be followed virtually. However, if assessment of tumor lesion is necessary (e.g., neoadjuvant breast cancer treatment), an in-person visit should be facilitated.	86%	-	-
C4c. Patients on surveillance/observation following definitive systemic therapy with curative intent can be followed virtually, unless symptoms arise on review of systems that trigger an in-person assessment. Engagement of locally accessible health care providers is recommended to arrange in-person physical examinations if indicated.	86%	-	-
C4d. Decisions to continue or discontinue systemic treatments that have been previously initiated could be made virtually, if deemed appropriate by health care provider and patient.	75%	-	100% (*n* = 17)
Survivorship			
C5a. Cancer survivors under surveillance following curative intent treatment can be safely followed using virtual platforms unless physical examination is indicated and/or required.	100%	-	-
C5b. Virtual inclusion and engagement with family medicine providers can be considered to optimize surveillance.	86%	-	-
C5c. Primary care providers and cancer survivors should be *formally* notified, in a survivorship care plan or similar document, of the transition to virtual survivorship care.	89%	-	-
Remote and Rural Communities			
C6a. Virtual care could be used for urgent consultation for distant patients who are either unable to visit a specialist in a timely manner or the severity of their symptoms prevent them from travelling long distances. Such visits could be accompanied by the attendance of local healthcare professionals (e.g., nurses, GPs, etc.).	96%	-	-

* Percentages are included if the statement was presented to the Consensus Group.

## Data Availability

Data is contained within the article and Supplementary Materials.

## Supplementary Materials

The following are available online at https://www.mdpi.com/article/10.3390/curroncol28060445/s1, Figure S1: Overview of systematic review results. Table S1: Key citations that informed first draft consensus statements.

## Appendix A. Questions Used by the Steering Committee to Draft Consensus Statements

Section A: Demographics, Logistics, and Implementation
  1. What are non-clinical patient characteristics that are best suited for virtual care?
  2. What environmental and equipment requirements are needed for virtual cancer care?
  3. How can virtual cancer care be implemented in multidisciplinary care?
  4. Does having access to local health care resources influence suitability for virtual care? What type of resources?
Section B: Diagnosis and Prognosis
  1. What are steps to facilitate safe and accurate diagnosis of cancer via virtual care?
  2. How can you best communicate a diagnosis, investigations (e.g., results of staging, blood tests) and prognosis virtually? Should diagnosis of cancer ever be made virtually?
  3. In what scenarios should a diagnosis or prognosis not be communicated virtually?
Section C: Clinical characteristics, active management, and follow-up
  1. What are the general recommendations that apply to the management of patients with cancer using virtual care?
  2. Are there particular clinical characteristics that are more conducive to virtual care?
  3. Which components of cancer-related surgery are suitable for virtual care?
  4. Which components of radiotherapy are suitable for virtual care?
  5. Which systemic treatment regimens can be managed using virtual care?
  6. What are cancer survivorship considerations during virtual care?
  7. What are rural and remote oncology considerations for virtual care?
