# Supplementary material for: Delivery of Virtual Care in Oncology: Province-Wide Interprofessional Consensus Statements Using a Modified Delphi Process
Source: Curr Oncol. 2021 Dec 13;28(6):5332–45. doi: 10.3390/curroncol28060445 (PMC8700064; doi:10.3390/curroncol28060445)
Supplement: Supplementary file 1 [file curroncol-28-00445-s001.zip › curroncol-1434287-supplementary.pdf]

Supplementary Materials

Consensus in Virtual Cancer Care

Matthew C. Cheung, Bryan B. Franco, Nicholas Meti, Alia Thawer, Houman Tahmasebi, Adithya Shankar, Andrew Loblaw, Frances C. Wright, Colleen Fox, Naomi Peek, Vivian Sim and Simron Singh on behalf of the Ontario Health (Cancer Care Ontario) Virtual Care Consensus Group

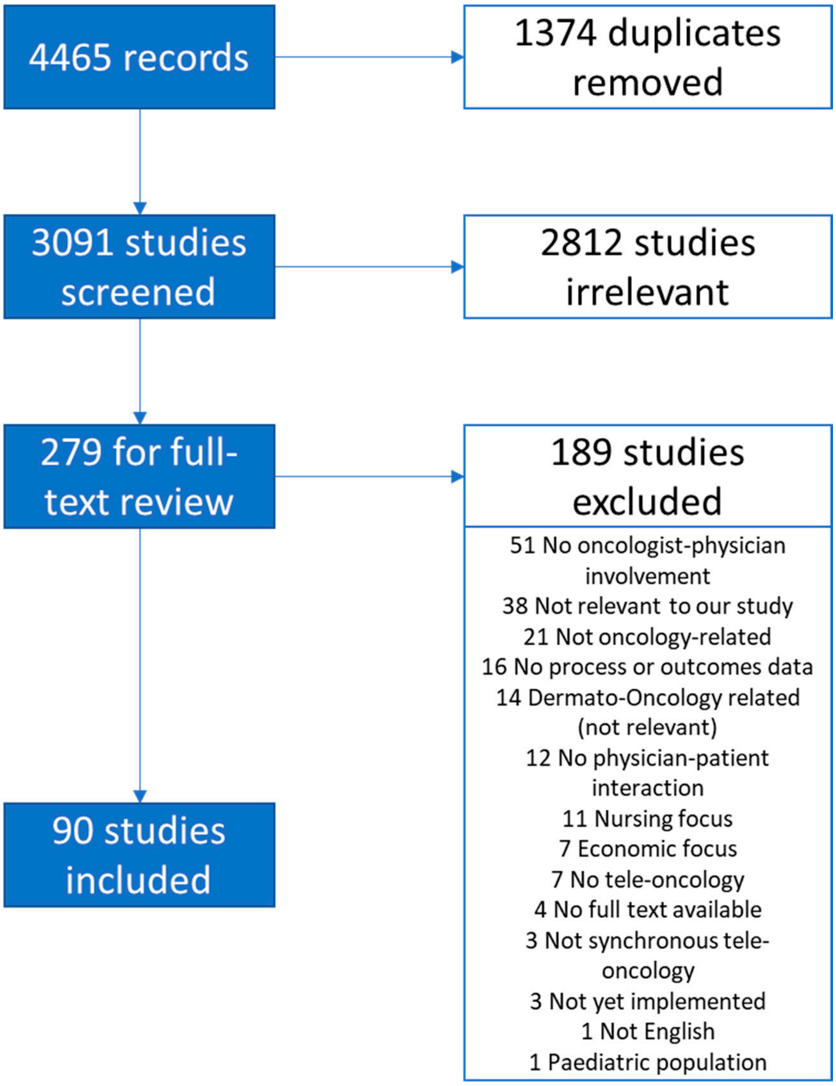

Figure S1. Overview of systematic review results. Full and updated results of the systematic review and an update will be published separately.

Table S1. Key citations that informed first draft consensus statements.

Section	Key Citations
**A: Demographics, logistics, and implementation**	Gurupur V, Shettian K, Xu P, et al. Identifying the readiness of patients in implementing telemedicine in northern Louisiana for an oncology practice. *Health informatics journal*. 2017;23(3):181–196.

	Sabesan S, Allen D, Loh PK, et al. Practical aspects of telehealth: Are my patients suited to telehealth? *Internal Medicine Journal.* 2013;43(5):581–584.
	Cartmill B, Wall LR, Ward EC, Hill AJ, Porceddu SV. Computer Literacy and Health Locus of Control as Determinants for Readiness and Acceptability of Telepractice in a Head and Neck Cancer Population. *International journal of telerehabilitation.* 2016;8(2):49–60.
	Brigden M, Minty A, Pilatzke S, Della Vidova L, Sherrington L, McPhail K. A survey of recipient client physician satisfaction with teleoncology services originating from Thunder Bay Regional Health Sciences Centre. *Telemedicine and e-Health.* 2008;14(3):250–254.
	Larcher B, Berloffa F, Demichelis F, et al. An evaluation of the use of and user satisfaction with a teleconsultation system in oncology practice. *Journal of Telemedicine and Telecare.* 2002;8 Suppl 2:28–30.
	Tarver WL, Haggstrom DA. The Use of Cancer-Specific Patient-Centered Technologies Among Underserved Populations in the United States: Systematic Review. *Journal of Medical Internet Research.* 2019;21(4):e10256.
	Stalfors J, Edstrom S, Bjork-Eriksson T, Mercke C, Nyman J, Westin T. Accuracy of tele-oncology compared with face-to-face consultation in head and neck cancer case conferences. *Journal of Telemedicine and Telecare.* 2001;7(6):338–343.
	Stalfors J, Holm-Sjogren L, Schwieler A, Tornqvist H, Westin T. Satisfaction with telemedicine presentation at a multidisciplinary tumour meeting among patients with head and neck cancer. *Journal of Telemedicine and Telecare.* 2003;9(3):150–155.
	Trabjerg TB, Jensen LH, Sondergaard J, Sisler JJ, Hansen DG. Improving continuity by bringing the cancer patient, general practitioner and oncologist together in a shared video-based consultation - protocol for a randomised controlled trial. *BMC family practice.* 2019;20(1):86.
	Jhaveri D, Larkins S, Sabesan S. Telestroke, tele-oncology and teledialysis: a systematic review to analyse the outcomes of active therapies delivered with telemedicine support. *Journal of Telemedicine and Telecare.* 2015;21(4):181–188.
**B: Diagnosis and Prognosis**	Saraswathula A, Lee JY, Megwalu UC. Patient preferences regarding the communication of biopsy results in the general otolaryngology clinic. *American Journal of Otolaryngology - Head and Neck Medicine and Surgery.* 2019;40(1):83–88.
	Patel MI, Periyakoil VS, Blayney DW, et al. Redesigning cancer care delivery: Views from patients and caregivers. *Journal of Oncology Practice.* 2017;13(4):e291–e302.
	McElroy JA, Proulx CM, Johnson LS, et al. Breaking bad news of a breast cancer diagnosis over the telephone: an

	emerging trend. *Supportive Care in Cancer.* 2019;27(3):943–950.<br>Sabesan S, Allen D, Caldwell P, et al. Practical aspects of telehealth: Doctor-patient relationship and communication. *Internal Medicine Journal.* 2014;44(1):101–103.<br>Fitzpatrick D, Grabarz D, Wang L, et al. How effective is a virtual consultation process in facilitating multidisciplinary decision-making for malignant epidural spinal cord compression? *International Journal of Radiation Oncology Biology Physics.* 2012;84(2):e167–e172.
**C: Clinical characteristics, active management, and follow-up**	Zhou M, Holden L, Bedard G, et al. The utilization of telephone follow-up in the advanced cancer population: A review of the literature. *Journal of Comparative Effectiveness Research.* 2012;1(6):509–517.<br>Rising KL, Ward MM, Goldwater JC, Bhagianadh D, Hollander JE. Framework to Advance Oncology-Related Telehealth. *JCO Clinical Cancer Informatics.* 2018;2:1–11.<br>Hamilton E, Van Veldhuizen E, Brown A, Brennan S, Sabesan S. Telehealth in radiation oncology at the Townsville Cancer Centre: Service evaluation and patient satisfaction. *Clinical and Translational Radiation Oncology.* 2019;15:20–25.<br>Sabesan S, Senko C, Schmidt A, et al. Enhancing chemotherapy capabilities in rural hospitals: Implementation of a telechemotherapy model (QReCS) in North Queensland, Australia. *Journal of Oncology Practice.* 2018;14(7):e429–e437.<br>Patil VM, Pande N, Chandrasekharan A, et al. Shadow study: randomized comparison of clinic with video follow-up in glioma undergoing adjuvant temozolomide therapy. *CNS oncology.* 2018;7(2):CNS14.<br>Shalowitz DI, Smith AG, Bell MC, Gibb RK. Teleoncology for gynecologic cancers. *Gynecologic Oncology.* 2015;139(1):172–177.<br>Chan BA, Larkins SL, Evans R, Watt K, Sabesan S. Do teleoncology models of care enable safe delivery of chemotherapy in rural towns? *Medical Journal of Australia.* 2015;203(10):406.<br>Weinerman B, den Duyf J, Hughes A, Robertson S. Can subspecialty cancer consultations be delivered to communities using modern technology?--A pilot study. *Telemedicine journal and e-health : the official journal of the American Telemedicine Association.* 2005;11(5):608–615.<br>Liptrott S, Bee P, Lovell K. Acceptability of telephone support as perceived by patients with cancer: A systematic review. *European Journal of Cancer Care.* 2018;27(1).<br>Agochukwu NQ, Skolarus TA, Wittmann D. Telemedicine and prostate cancer survivorship: a narrative review. *mHealth.* 2018;4:45.

Dickinson R, Hall S, Sinclair JE, Bond C, Murchie P. Using technology to deliver cancer follow-up: a systematic review. *BMC Cancer.* 2014;14:311.

Qaderi SM, Vromen H, Dekker HM, Stommel MWJ, Bremers AJA, de Wilt JHW. Development and implementation of a remote follow-up plan for colorectal cancer patients. *European Journal of Surgical Oncology.* 2020;46(3):429–432.

Pruthi S, Stange KJ, Malagrino GD, Jr., Chawla KS, LaRusso NF, Kaur JS. Successful implementation of a telemedicine-based counseling program for high-risk patients with breast cancer. *Mayo Clinic Proceedings.* 2013;88(1):68–73.

Sirintrapun SJ, Lopez AM. Telemedicine in Cancer Care. *Am Soc Clin Oncol Educ Book.* 2018;38:540–545.

Sabesan S, Larkins S, Evans R, et al. Telemedicine for rural cancer care in North Queensland: bringing cancer care home. *The Australian journal of rural health.* 2012;20(5):259–264.

Nawas MT, Landau HJ, Sauter CS, et al. Pilot Study of Telehealth Evaluations in Patients Undergoing Hematopoietic Cell Transplantation. *Biology of Blood and Marrow Transplantation.* 2020.

Beswick DM, Vashi A, Song Y, et al. Consultation via telemedicine and access to operative care for patients with head and neck cancer in a Veterans Health Administration population. *Head & neck.* 2016;38(6):925–929.

Agboola SO, Ju W, Elfiky A, Kvedar JC, Jethwani K. The effect of technology-based interventions on pain, depression, and quality of life in patients with cancer: a systematic review of randomized controlled trials. *Journal of Medical Internet Research.* 2015;17(3):e65.

Katz MHG, Slack R, Bruno M, et al. Outpatient virtual clinical encounters after complex surgery for cancer: A prospective pilot study of "teleDischarge". *Journal of Surgical Research.* 2016;202(1):196–203.

Barsom EZ, Jansen M, Tanis PJ, et al. Video consultation during follow up care: effect on quality of care and patient- and provider attitude in patients with colorectal cancer. *Surgical Endoscopy.* 2020.

Ferrari R, Amouzou KS, Cobitti C, Bartolo M. Teleoncology in sub-Saharan Africa: A literature review. *Journal of Cancer Policy.* 2018;17:9–14.

Doolittle GC, Allen A. Practising oncology via telemedicine. *J Telemed Telecare.* 1997;3(2):63–70.

Weinerman BH, Barnett J, Loyola M, et al. Telehealth--a change in a practice model in oncology. *Telemedicine journal and e-health : the official journal of the American Telemedicine Association.* 2012;18(5):391–393.

	Kunkler IH, Rafferty P, Hill D, Henry M, Foreman D. A pilot study of tele-oncology in Scotland. *Journal of Telemedicine and Telecare.* 1998;4(2):113–119.
	Greenhalgh T, Shaw S, Wherton J, et al. Real-World Implementation of Video Outpatient Consultations at Macro, Meso, and Micro Levels: Mixed-Method Study. *Journal of Medical Internet Research.* 2018;20(4):e150.
	Sabesan S, Simcox K, Marr I. Medical oncology clinics through videoconferencing: an acceptable telehealth model for rural patients and health workers. *Intern Med J.* 2012;42(7):780–785.
	Haghighat S, Yunesian M, Akbari ME, Ansari M, Montazeri A. Telephone and face-to-face consultation in breast cancer diagnosis: A comparative study. *Patient Education and Counseling.* 2007;67(1–2):39–43.
	Cox A, Lucas G, Marcu A, et al. Cancer Survivors' Experience With Telehealth: A Systematic Review and Thematic Synthesis. *Journal of Medical Internet Research.* 2017;19(1):e11.
	Galsky MD, Shahin M, Jia R, et al. Telemedicine-Enabled Clinical Trial of Metformin in Patients With Prostate Cancer. *JCO Clinical Cancer Informatics.* 2017;1:1–10.
	Novoa NM, Gomez MT, Rodriguez M, et al. e-Consultation Improves Efficacy in Thoracic Surgery Outpatient Clinics. *La discusion de casos por videoconferencia mejora la eficiencia de la consulta externa de cirugia toracica.* 2016;52(11):549–552.
